# Effect of CCR7, CXCR4 and VEGF-C on the lymph node metastasis of human pancreatic ductal adenocarcinoma

**DOI:** 10.3892/ol.2013.1261

**Published:** 2013-03-15

**Authors:** JINGHUI GUO, WENHUI LOU, YUAN JI, SHUNCAI ZHANG

**Affiliations:** 1 Department of Gastroenterology, Sixth People’s Hospital Affiliated to Shanghai Jiao Tong University, Shanghai, P.R. China; 2 Departments of General Surgery, Zhongshan Hospital Affiliated to Fudan University, Shanghai, P.R. China; 3 Pathology, Zhongshan Hospital Affiliated to Fudan University, Shanghai, P.R. China; 4 Gastroenterology, Zhongshan Hospital Affiliated to Fudan University, Shanghai, P.R. China

**Keywords:** CCR7, CXCR4, VEGF-C, pancreatic ductal adenocarcinoma, lymph node metastasis

## Abstract

The aim of the present study was to investigate the association between the expression of chemokine receptors CCR7 and CXCR4 and vascular endothelial growth factor (VEGF)-C and the lymph node metastasis of pancreatic ductal adenocarcinoma (PDAC). The mRNA transcription levels of CCR7, CXCR4 and VEGF-C were measured in 24 specimens by real-time reverse transcription (RT)-PCR, while the protein expression levels were measured in 65 specimens by immuohistochemistry. Professional software for pathological image manipulation (Image Pro Plus 6.0) was used to quantitate the results of the immunohistochemical staining. The mRNA and protein expression levels of CCR7, CXCR4 and VEGF-C were all significantly higher in the cancer samples compared with those in the adjacent normal tissue. The CCR7 and VEGF-C mRNA and protein expression levels were significantly higher in the patients with cancer types exhibiting lymph node metastasis and an advanced International Union Against Cancer (UICC) stage (P<0.05). The greater the number of metastatic lymph nodes, the higher the levels of CCR7 expression (P<0.05). There was a significant positive linear correlation between the mRNA and protein expression levels of CCR7 and VEGF-C (P<0.05). The mRNA and protein expression levels of CXCR4 were not correlated with the lymph node metastasis (P>0.05), however the strong positive expression of CCR7 and VEGF-C was significantly associated with the lymph node metastasis of PDAC.

## Introduction

The survival rate of patients with pancreatic ductal adenocarcinoma (PDAC) is worse than that of patients with other gastrointestinal malignancies. The principal reasons for this poor prognosis include difficulties in diagnosing PDAC at a localized resectable stage and the propensity of the tumor for early metastasis to the regional lymph nodes and liver. The presence or absence of lymph node metastasis is one of the key prognostic factors for patients with PDAC. Therefore, an assessment of the status of the regional lymph nodes is required to affect the survival outcomes and therapeutic methods of choice (1,2).

However, the mechanisms by which tumor cells detach from the primary tumor, invade lymphatic vessels and metastasize to regional lymph nodes are complex. Previous data has suggested that chemokine receptors may direct the lymphatic spread and additionally affect the sites of the metastatic growth of various tumors (3). Originally, chemokines and their G protein-coupled receptors were reported to mediate various pro- and anti-inflammatory responses. CCR7, the receptor for the chemokine CCL21, is expressed on naive T cells, memory T cells, B cells and mature dendritic cells, and is considered to be important in lymphocyte cell trafficking and homing to the lymph nodes (4,5). The chemokine receptor CXCR4 was initially described as being able to regulate the homing of lymphocytes in inflammatory tissues (6). The natural ligand of CXCR4, stromal cell-derived factor 1a (SDF-1a), is highly expressed in tissues of metastatic growth, including those of the lung, liver and lymph nodes, and also attracts lymphocytes to these organs (7). Vascular endothelial growth factor (VEGF)-C, a member of the VEGF family, has been reported to be a lymphatic-specific growth factor (8,9). VEGF-C is the first ligand to be identified for VEGFR-3 (8). Since the expression of VEGFR-3 is predominantly restricted to the lymphatic endothelium in adults (10), the major function of VEGF-C appears to be the regulation of lymphatic vessel growth.

These two chemokine receptors, along with VEGF-C, have been brought into focus with regard to their role in the spreading of tumors. CCR7 expression has been shown to be positively correlated with lymphatic metastasis and a poor prognosis in squamous cell, oral and oropharyngeal squamous cell carcinoma and breast, colorectal, esophageal and prostate cancers (11–15). High CXCR4 expression has been associated with lymph node metastases in breast cancer and oral squamous cell carcinoma (16,17). The levels of VEGF-C in primary tumors have been significantly correlated with lymph node metastasis in a variety of cancer types, including oral squamous cell cancer, squamous cell carcinomas of the head and neck, non-small cell lung carcinoma, cervical cancer and colorectal cancer (18–22). Gastric cancer, which exhibited a co-expression of CCR7 and CXCR4, was revealed to be more likely to include lymph node metastasis (23). High VEGF-C and CXCR4 expression levels in hepatocellular carcinoma have also been associated with lymph node metastasis (24). It is possible that CCR7, CXCR4 and VEGF-C interact with each other in the process of the metastatic spread of tumor cells to distant regional lymph nodes.

However, no data are currently available with regard to the co-expression of CCR7, CXCR4 and VEGF-C in PDAC and their association with each other. Therefore, the present study evaluated the mRNA and protein expression levels of CCR7, CXCR4 and VEGF-C in PDAC and correlated the results with the patients’ clinicopathological parameters.

## Materials and methods

### Patients and tissue specimens

Tumor tissue was collected for RNA extraction from 24 patients with PDAC who underwent curative surgery between 2006 and 2008, and for immunohistochemistry from 65 patients with PDAC who underwent curative surgery between 2004 and 2008 at the Department of Surgery, Zhongshan Affiliated Hospital of Fudan University (Shanghai, China). Written informed consent was obtained from each individual. The study was approved by the biomedical research Ethics Committee of Affiliated Zhongshan Hospital of Fudan University (Shanghai, China). Patients were excluded if they had received neoadjuvant chemotherapy or radiotherapy. The freshly removed PDAC tissues for RNA extraction were immediately frozen in liquid nitrogen and stored at −80°C until further use. The baseline characteristics of these patients are shown in Table I. The tissue samples that were to be used for immunohistochemistry and HE staining were fixed in formalin and then embedded in paraffin. The baseline characteristics of these patients are shown in Table II.

### Real-time reverse transcription (RT)-PCR analysis

Real-time RT-PCR tumor tissue blocks of ∼1×1×1 cm, wrapped in silver paper, were rapidly frozen in liquid nitrogen for ∼1 min, then stored in a −80°C refrigerator until the target mRNA was detected. Total RNA was purified from fresh soft tissues using TRIzol according to the manufacturer’s instructions. The purity of the RNA was measured and determined with a UV spectrophotometer and the OD 260/280 value was 1.8–2.1. To normalize the expressed cytokine mRNA, the internal housekeeping β-actin gene was used. The gene transcription of CCR7, CXCR4, VEGF-C and β-actin was analyzed by two-step RT-PCR. Reverse transcription was performed with 500 ng RNA (10 *μ*l total volume; SYBR^®^ PrimeScript™ RT-PCR kit, Takara Biotechnology Co. Ltd., Dalian, China) according to the manufacturer’s instructions. The first-standard cDNA solution was used as a template for the specific PCR reactions. The primers used were as follows: CCR7 sense, 5′-CTCCAGGCACGCAACTTTGA-3′ and antisense, 5′-CACAGGTGCTACTGGTGATGTTGA-3′ (145-bp fragment); CXCR4 sense, 5′-GCCAACGTCAGTGAGGCAGA-3′ and antisense, 5′-GCCAACCATGATGTGCTGAAAC-3′ (99-bp fragment); VEGF-C sense, 5′-CAGCACGAGCTACCTCAGCAAG-3′ and antisense, 5′-TTTAGACATGCATCGGCAGGAA-3′ (115-bp fragment); and β-actin sense, 5′-TGAGATGCGTTGTTACAGGA-3′ and antisense, 5′-ACGAAAGCAATGCTATCACC-3′ (119-bp fragment). The cycling conditions of the PCRs were as follows: An initial denaturation for 10 sec at 95°C, followed by 40 cycles of denaturation for 5 sec at 95°C and annealing for 30 sec at 60°C. Following the last cycle, a final extension of 10 sec at 60°C was completed and thereafter the samples were maintained at 4°C. The products (15 *μ*l) were run on a 2.5% agarose gel, stained with ethidium bromide and analyzed under UV light.

Quantitative real-time RT-PCR was performed with an IQ5TM Sequence Detection System (Bio-Rad, Hercules, CA, USA), according to the manufacturer’s instructions. The reaction mixture was composed of SYBR Green Mastermix, 12.5 *μ*l of each primer and cDNA with a total volume of 25 *μ*l. For each gene, a standard curve was used to calculate the gene expression levels, thus correcting for the different primer efficiencies. To calculate the data, the comparative C_t_ method (^2−ΔΔCt^ method) was used for the relative quantification, which described the change in the expression of the target gene in a test sample and provided accurate comparisons with the initial levels of the template in each sample. The data were analyzed with SPSS software version 17.0.

### Immunohistochemistry and staining evaluation

Immunohistochemical studies were performed for comparison with the results from the mRNA expression analysis obtained by real-time RT-PCR. The paraffin-embedded tissue sections (3–5-*μ*m thick) were subjected to immunostaining for CCR7, CXCR4 (EliVision™ plus kit; Maixin Bio, Fuzhou, China) and VEGF-C (EliVision™ plus kit; ZSGB Bio Systems, Beijing, China). The sections were mounted on positively charged slides, incubated for 45 min at 60°C and deparaffinized. Antigen retrieval was performed with a microwave to boil the tissue sections in 10 mM sodium citrate buffer (pH 6.0) for 20 min. Subsequent to the endogenous peroxidase activity being blocked with a 3% aqueous H_2_O_2_ solution for 10 min, the tissue was incubated with the primary antibodies for CCR7 (ab32527, rabbit monoclonal IgG; Abcam Cambridge Chemical Co., Cambridge, MA, USA) at a 1:200 dilution, for CXCR4 (ab2074, rabbit polyclonal IgG Abcam Cambridge Chemical Co.) and VEGF-C (AF752, goat polyclonal IgG; R&D Systems, Minneapolis, MN, USA) at 1:100 dilution respectively, for 1 h. The tissue slides were then incubated with a reinforcing agent for 20 min. The slides were rinsed with washing buffer and color was developed with a DAB detection kit (Maixin Bio, Fuzhou, China) following incubation with the anti-rabbit antibodies for CCR7 and CXCR4 and the anti-goat antibodies for VEGF-C. For the negative controls, PBS buffer was substituted for the primary antibody. The intensity, staining percentage and pattern of staining were assessed.

The immunostaining was evaluated in a manner that was blinded to the patient outcome and all clinicopathological findings. Quantification of the immunostaining was performed by digital image analysis with the Image-Pro Plus 6.0 software (Media Cybernetics, Inc., Silver Spring, MD, USA). This method uses areas of specific staining from the various images to determine the positivity discrimination plane, minimizing the possible visual variation in the detection of immunostained areas over time when an interactive discrimination plane is used. To count the total area, the discrimination plane was set at 0–30 in the H channel and 0–255 in the S and I channels.

**Figure 1:**
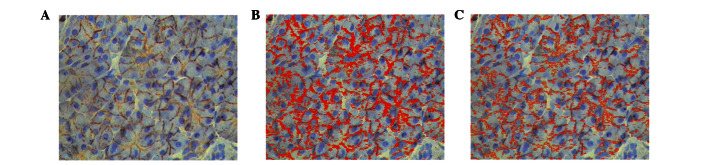
Quantification of immunostaining by digital image analysis. (A) Original field acquired from tissue sections (magnification, ×400). (B) Section marking of (A). The positive staining of the AOI was marked in red. (C) Area of positive staining marked by red lines. (B) Marked tumoral positivity and (C) total area were counted through digital image analysis. The resulting mean density was expressed as an index (IOD/AOI). IOD, integrated optical density; AOI, area of interest.

Identical settings were used for each field. A total of five fields selected from hot-spot areas (400X objective lens) were acquired per slide. The integrated optical density (IOD) of all the positive staining in each field and area of interest (AOI) was measured. The IOD was used to evaluate the area and intensity of the positive staining. The mean density (IOD/AOI) represented the concentration of specific protein per unit area (Fig. 1A–C).

### Statistical analysis

SPSS 17.0 software (SPSS, Inc., Chicago, IL, USA) was used for the statistical analysis. Categorical variables were evaluated by the Wilcoxon rank-sum and Pearson’s correlation tests. P<0.05 was considered to indicate statistically significant differences.

## Results

### Expression of CCR7, CXCR4 and VEGF-C mRNA and clinicopathological factors

**Figure 2:**
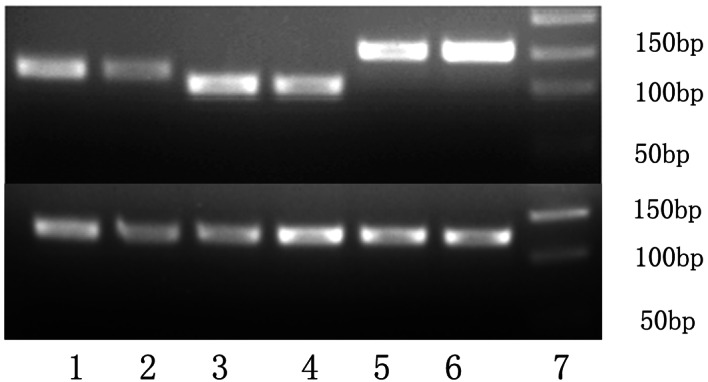
Agarose gel electrophoresis of RT-PCR-amplified 145-bp CCR7, 99-bp CXCR4 and 115-bp VEGF-C cDNA, with 119-bp β-actin cDNA as the internal PCR control. Top gel, objective gene; bottom gel, internal control β-actin gene. Lane 7, size marker; lanes 1 and 2, adenocarcinoma with positive VEGF-C expression; lanes 3 and 4, adenocarcinoma with positive CXCR4 expression; lanes 5 and 6, adenocarcinoma with positive CCR7 expression. VEGF, vascular endothelial growth factor.

A total of 24 PDAC specimens and 24 adjacent normal tissues were examined for the expression of CCR7, CXCR4 and VEGF-C mRNA (Fig. 2). The expression levels of CCR7, CXCR4 and VEGF-C mRNA in the cancer samples were all significantly higher than those in the adjacent normal tissue. There were no significant differences between the CCR7, CXCR4 and VEGF-C mRNA expression levels and the age, gender or tumor grading (P>0.05). However, the expression of the CCR7 and VEGF-C mRNA was significantly correlated with lymph node metastasis and the advanced International Union Against Cancer (UICC) stage (P=0.001 for lymph node metastasis and for staging). However, the correlation between the expression of CXCR4 mRNA and lymph node metastasis or UICC stage was not statistically significant (P>0.05).

### Correlations among the expression of CCR7, CXCR4 and VEGF-C mRNA

Spearman’s rank correlation analyses showed that there were correlations among the mRNA expression levels of CCR7, CXCR4 and VEGF-C. There was a significant positive linear correlation between the expression levels of CCR7 and VEGF-C (r=0.915, P<0.001), but not between CCR7 and CXCR4 or CXCR4 and VEGF-C (P>0.05).

### Expression of CCR7, CXCR4 and VEGF-C protein, and clinicopathological factors

**Figure 3:**
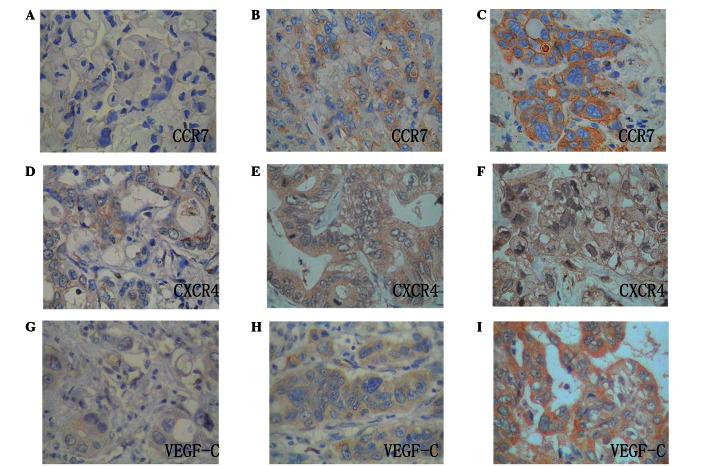
Intensity of CCR7, CXCR4 and VEGF-C expression in patients with PDAC (magnification, ×400). (A–C) Expression grades of CCR7 in the cytoplasm and cell membrane: (A) Weak expression; (B) intermediate expression; and (C) marked expression. (D–F) Expression grades of CXCR4 in the cytoplasm and cell nucleus: (D) Weak expression; (E) intermediate expression; and (F) marked expression. (G–I) Expression grades of VEGF-C in the cytoplasm: (G) Weak expression; (H) intermediate expression; and (I) marked expression. PDAC, pancreatic ductal adenocarcinoma; VEGF, vascular endothelial growth factor.

Staining for the CCR7 protein was identified in the cytoplasm and cell membrane of the cancer cells and was not detected in the cytoplasm of the normal pancreatic cells obtained from the non-cancerous regions of the PDAC tissue. Staining for the CXCR4 protein was also identified in the cytoplasm and cell nucleus of the cancer cells, but not in the cell nucleus of the normal pancreatic cells obtained from the non-cancerous regions of the PDAC tissue. The immunohistological localization of VEGF-C was cytoplasmic in the cancer and normal pancreatic cells obtained from the non-cancerous regions of the PDAC tissue. In the cancer specimens with positive expression, the number of immunoreactive cells ranged from a few to almost all of the tumor cells (Fig. 3).

The expression levels of CCR7, CXCR4 and VEGF-C in the cancer cells were all significantly higher than those in the non-cancerous regions (P<0.05). Furthermore, the patients with a higher CCR7 and VEGF-C expression in their cancer cells had significantly higher incidences of lymphatic metastasis (P<0.01). The greater the number of metastatic lymph nodes, the higher the level of CCR7 expression. Patients with a higher CCR7 and/or VEGF-C expression also had a higher UICC stage (P<0.05). However, the correlation between the expression of the CXCR4 protein and lymph node metastasis or UICC was not statistically significant (P>0.05). The expression levels of CCR7, CXCR4 and VEGF-C did not correlate with the clinicopathological parameters, including those of age, gender, tumor size and histological grade (P>0.05).

### Correlations among the expression levels of CCR7, CXCR4 and VEGF-C protein

Spearman’s rank correlation analyses showed that there were correlations among the levels of CCR7, CXCR4 and VEGF-C expression. There were significant positive linear correlations between the expression levels of CCR7 and CXCR4 or VEGF-C (r=0.449, P<0.001; r=0.770, P<0.05), but not between CXCR4 and VEGF-C (P>0.05).

## Discussion

PDAC is a type of malignant tumor with marked invasive characteristics and a high incidence of lymph node metastasis. Of the patients with small cell PDAC, 30–40% have lymph node micrometastases which may be observed by immunohistochemistry or other molecular biological methods (25). At present, surgery remains the primary treatment for resectable PDAC, although the effect of this surgery is not completely satisfactory. Previous clinical studies have shown that lymph node metastasis is the main cause of tumor recurrence following pancreaticoduodenectomy (1). Even patients who have undergone extended regional lymph node dissection may experience lymphatic metastatic recurrence (26,27).

Therefore, adopting a targeted adjuvant therapy to control lymphatic metastatic recurrence following surgery is one of the key approaches to improve the survival rate of patients with PDAC. The identification of post-operative factors associated with lymph node metastasis is likely to be of great clinical significance for the application of targeted adjuvant therapy. Usually, tumor prognosis is predicted from the TNM classification, although this modality lacks sensitivity and accuracy. Thus, identifying molecular biomarkers with the potential to predict the prognosis of PDAC may potentially compensate for the lack of efficacy of conventional methods.

It is well-known that tumor chemotactic migration and lymphangiogenesis are directly correlated with lymph node metastasis. A study by Müller *et al* revealed that tumor cells with CCR7-positive expression preferentially transfer to the lymph nodes that are rich in the ligand CCL21 (28). This provided a theoretical basis behind the phenomenon of certain tumor cells preferentially transferring to regional lymph nodes. Nakata *et al*(29) reported that the high expression of CCR7 was correlated with lymph node metastasis and a poor prognosis in PDAC. However, lymphangiogenesis is a precondition for lymph node metastasis. The current hypothesis is that VEGF-C may be expressed in a variety of solid tumors and that it is able to induce lymphatic endothelial cell mitosis by binding to the receptors VEGFR-3 and VEGFR-2, which are important in lymphangiogenesis. The expression level of VEGF-C is positively correlated with lymph node metastasis. Previous studies have shown that the expression of VEGF-C in PDAC is also closely correlated with lymph node metastasis (30,31). However, it is unknown whether the expression of CCR7 and VEGF-C are correlated with each other and if together they may cause lymph node metastasis in PDAC. Whether CXCR4 is involved in this process also remains unclear.

The present study is the first to detect the expression of CCR7, CXCR4 and VEGF-C in tumor tissues using real-time RT-PCR and immunohistochemistry assays in a large series of human PDAC specimens. It was demonstrated that the expression levels of CCR7, CXCR4 and VEGF-C mRNA and protein were all significantly higher in the cancer specimens compared with those in the adjacent normal tissue. The CCR7 and VEGF-C mRNA and protein expression levels were significantly higher in patients with cancer types exhibiting lymph node metastasis and a more advanced UICC stage. Furthermore, the greater the number of metastatic lymph nodes, the higher the level of CCR7 expression. There was a significant positive linear correlation between the mRNA and protein expression levels of CCR7 and VEGF-C. This indicates that CCR7 and VEGF-C mRNA and protein expression are upregulated in cases with a greater number of metastasis-positive nodes and may contribute to lymph node metastasis occurring in PDAC. The present data are consistent with previous studies that describe a positive correlation between CCR7 expression and lymph node metastasis in cases of breast, colorectal, esophageal and prostate cancer and oral and oropharyngeal squamous cell carcinoma (11–15). A positive correlation has also been reported between VEGF-C expression and lymph node metastasis in cases of oral squamous cell cancer, squamous cell carcinomas of the head and neck, non-small cell lung carcinoma, cervical cancer and colorectal cancer (18–22). Although the role of chemokines and their receptors in human cancers is complex, the chemokine receptors CCR7 and VEGF-C may be critical in determining lymph node metastasis in these types of tumors. Wehler *et al*(32) reported that marked CXCR4 expression was significantly associated with advanced UICC stages and also revealed a correlation with hematogenous metastasis. Studies have shown that CXCR4 is involved in pancreatic cancer progression through the promotion of angiogenesis (33). However, the present study showed that the correlation between the expression of CXCR4 and UICC was not statistically significant. In the present study, the samples were divided into two groups (I and IIA or IIB, III and IV) according to the UICC stage, which is different to the grouping method within the literatur. It is possible that patients with stage II PDAC had hematogenous metastasis. Thus, the difference of the result could be interpreted and at the same time it was conferred that CXCR4 did not play a key role in the lymph node metastasis of PDAC.

In accordance with previous studies conducted on other types of cancer, the staining for CCR7 was localized in the membrane and cytoplasm of the tumor cells, while VEGF-C was predominantly cytoplasmic. Staining for CCR7 was observed in the membrane of the normal cells. This may represent the functional status of the receptor since binding to a specific ligand induces receptor internalization. The necessity of internalization for chemotaxis and signaling remains controversial. Endosomes are gaining considerable attention as scaffolds for signaling complexes. The assembly of signaling complexes on intracellular endosomal membranes indicates that the intracellular trafficking itinerary of chemokine receptors may have significant implications for signaling (34).

In conclusion, surgery remains the primary treatment for PDAC in China. Although there are still a range of views with regard to whether patients who have undergone complete resection of PDAC should receive adjuvant therapy, due to the high incidence of lymphatic metastatic recurrence after pancreaticoduodenectomy, we recommend that patients at a high risk of lymphatic metastatic recurrence receive targeted adjuvant therapy following surgery. The present study suggested that the positive expression of CCR7 and VEGF-C are closely correlated with lymphatic metastatic recurrence in PDAC. Therefore, these two molecular indicators may become a reference index for the clinical assessment of lymphatic metastatic recurrence and poor outcome in patients who should receive additional treatment, including molecular targeted therapy and follow-up examinations following surgical treatment. The present study has laid the groundwork for research into the molecular mechanism and targeted adjuvant therapy for lymphatic metastases of PDAC. However, the limitations of the present study include the use of a small number of patients and limited research conditions, and consequently, the results do not permit final conclusions to be drawn. We plan to conduct a prospective study of a large number of cases to confirm these results.

## Figures and Tables

**Table I:** Baseline characteristics of patients whose samples were used for RNA extraction (n=24).

Variable	Value
Total, n	24
Median age, years (n ± SD)	63.87±11.22
Age range, years	42–80
Gender, n (%)	
Male	16 (67)
Female	8 (33)
Histological grade, n (%)	
Poorly differentiated	14 (58)
Well/moderately differentiated	10 (42)
Tumour stage, n (%)	
T1–T2	18 (75)
T3–T4	6 (25)
UICC stage, n (%)	
I + IIA	8 (33)
IIB + III + IV	16 (67)
Lymph node metastasis, n (%)	
Yes	16 (67)
No	8 (33)

UICC, advanced International Union Against Cancer stage.

**Table II:** Baseline characteristics of patients whose samples were used for immunohistochemistry (n=65).

Variable	Value
Total, n	65
Median age, years (n ± SD)	63.18±9.23
Age range, years	44–81
Gender, n (%)	
Male	49 (75)
Female	16 (25)
Histological grade, n (%)	
Poorly differentiated	33 (51)
Well/moderately differentiated	32 (49)
Tumour stage, n (%)	
T1–T2	60 (92)
T3–T4	5 (8)
UICC stage, n (%)	
I + IIA	39 (60)
IIB + III + IV	26 (40)
Lymph node metastasis, n (%)	
Yes	27 (42)
No	38 (58)

UICC, advanced International Union Against Cancer stage.

## References

[1] Schnelldorfer T, Ware AL, Sarr MG, Smyrk TC, Zhang L, Qin R, Gullerud RE, Donohue JH, Nagorney DM, Farnell MB (2008). Long-term survival after pancreatoduodenectomy for pancreatic adenocarcinoma: is cure possible?. Ann Surg.

[2] House MG, Gönen M, Jarnagin WR (2007). Prognostic significance of pathologic nodal status in patients with resected pancreatic cancer. J Gastrointest Surg.

[3] Arya M, Patel HR, Williamson M (2003). Chemokines: key players in cancer. Curr Med Res Opin.

[4] Dieu MC, Vanbervliet B, Vicari A, Bridon JM, Oldham E, Aït-Yahia S, Brière F, Zlotnik A, Lebecque S, Caux C (1998). Selective recruitment of immature and mature dendritic cells by distinct chemokines expressed in different anatomic sites. J Exp Med.

[5] Hirao M, Onai N, Hiroishi K, Watkins SC, Matsushima K, Robbins PD, Lotze MT, Tahara H (2000). CC chemokine receptor-7 on dendritic cells is induced after interaction with apoptotic tumor cells: critical role in migration from the tumor site to draining lymph nodes. Cancer Res.

[6] Murdoch C (2000). CXCR4: chemokine receptor extraordinaire. Immunol Rev.

[7] Phillips RJ, Burdick MD, Lutz M, Belperio JA, Keane MP, Strieter RM (2003). The stromal derived factor-1/CXCL12-CXC chemokine receptor 4 biological axis in non-small cell lung cancer metastases. Am J Respir Crit Care Med.

[8] Joukov V, Pajusola K, Kaipainen A, Chilov D, Lahtinen I, Kukk E, Saksela O, Kalkkinen N, Alitalo K (1996). A novel vascular endothelial growth factor, VEGF-C, is a ligand for the Flt4 (VEGFR-3) and KDR (VEGFR-2) receptor tyrosine kinases. EMBO J.

[9] Orlandini M, Marconcini L, Ferruzzi R, Oliviero S (1996). Identification of a c-fos-induced gene that is related to the platelet-derived growth factor/vascular endothelial growth factor family. Proc Natl Acad Sci USA.

[10] Kaipainen A, Korhonen J, Mustonen T, van Hinsbergh VW, Fang GH, Dumont D, Breitman M, Alitalo K (1995). Expression of the fms-like tyrosine kinase FLT4 gene becomes restricted to lymphatic endothelium during development. Proc Natl Acad Sci USA.

[11] Cabioglu N, Yazici MS, Arun B, Broglio KR, Hortobagyi GN, Price JE, Sahin A (2005). CCR7 and CXCR4 as novel biomarkers predicting axillary lymph node metastasis in T1 breast cancer. Clin Cancer Res.

[12] Günther K, Leier J, Henning G (2005). Predictor of lymph node metastasis in colorectal carcinoma by expression of chemokine receptor CCR7. Int J Cancer.

[13] Ding Y, Shimada Y, Maeda M, Kawabe A, Kaganoi J, Komoto I, Hashimoto Y, Miyake M, Hashida H, Imamura M (2003). Association of CC chemokine receptor 7 with lymph node metastasis of esophageal squamous cell carcinoma. Clin Cancer Res.

[14] Heresi GA, Wang J, Taichman R, Chirinos JA, Regalado JJ, Lichtstein DM, Rosenblatt JD (2005). Expression of the chemokine receptor CCR7 in prostate cancer presenting with generalized lymphadenopathy: report of a case, review of the literature, and analysis of chemokine receptor expression. Urol Oncol.

[15] Tsuzuki H, Takahashi N, Kojima A, Narita N, Sunaga H, Takabayashi T, Fujieda S (2006). Oral and oropharyngeal squamous cell carcinomas expressing CCR7 have poor prognoses. Auris Nasus Larynx.

[16] Kato M, Kitayama J, Kazama S, Nagawa H (2003). Expression pattern of CXC chemokine receptor-4 is correlated with lymph node metastasis in human invasive ductal carcinoma. Breast Cancer Res.

[17] Uchida D, Begum NM, Almofti A, Nakashiro K, Kawamata H, Tateishi Y, Hamakawa H, Yoshida H, Sato M (2003). Possible role of stromal-cell-derived factor-1/CXCR4 signaling on lymph node metastasis of oral squamous cell carcinoma. Exp Cell Res.

[18] Sedivy R, Beck-Mannagetta J, Haverkampf C, Battistutti W, Hönigschnabl S (2003). Expression of vascular endothelial growth factor-C correlates with the lymphatic microvessel density and the nodal status in oral squamous cell cancer. J Oral Pathol Med.

[19] Neuchrist C, Erovic BM, Handisurya A, Fischer MB, Steiner GE, Hollemann D, Gedlicka C, Saaristo A, Burian M (2003). Vascular endothelial growth factor C and vascular endothelial growth factor receptor 3 expression in squamous cell carcinomas of the head and neck. Head Neck.

[20] Arinaga M, Noguchi T, Takeno S, Chujo M, Miura T, Uchida Y (2003). Clinical significance of vascular endothelial growth factor C and vascular endothelial growth factor receptor 3 in patients with nonsmall cell lung carcinoma. Cancer.

[21] Van Trappen PO, Steele D, Lowe DG, Baithun S, Beasley N, Thiele W, Weich H, Krishnan J, Shepherd JH, Pepper MS, Jackson DG, Sleeman JP, Jacobs IJ (2003). Expression of vascular endothelial growth factor (VEGF)-C and VEGF-D, and their receptor VEGFR-3, during different stages of cervical carcinogenesis. J Pathol.

[22] Hanrahan V, Currie MJ, Gunningham SP, Morrin HR, Scott PA, Robinson BA, Fox SB (2003). The angiogenic switch for vascular endothelial growth factor (VEGF)-A, VEGF-B, VEGF-C, and VEGF-D in the adenoma-carcinoma sequence during colorectal cancer progression. J Pathol.

[23] Arigami T, Natsugoe S, Uenosono Y, Yanagita S, Arima H, Hirata M, Ishigami S, Aikou T (2009). CCR7 and CXCR4 expression predicts lymph node status including micrometastasis in gastric cancer. Int J Oncol.

[24] Xiang Z, Zeng Z, Tang Z, Fan J, Sun H, Wu W, Tan Y (2009). Increased expression of vascular endothelial growth factor-C and nuclear CXCR4 in hepatocellular carcinoma is correlated with lymph node metastasis and poor outcome. Cancer J.

[25] Nakao A, Takeda S, Sakai M, Kaneko T, Inoue S, Sugimoto H, Kanazumi N (2004). Extended radical resection versus standard resection for pancreatic cancer: the rationale for extended radical resection. Pancreas.

[26] Büchler MW, Kleeff J, Friess H (2007). Surgical treatment of pancreatic cancer. J Am Coll Surg.

[27] Koliopanos A, Avgerinos C, Farfaras A, Manes C, Dervenis C (2008). Radical resection of pancreatic cancer. Hepatobiliary Pancreat Dis Int.

[28] Müller A, Homey B, Soto H, Ge N, Catron D, Buchanan ME, McClanahan T, Murphy E, Yuan W, Wagner SN, Barrera JL, Mohar A, Verástegui E, Zlotnik A (2001). Involvement of chemokine receptors in breast cancer metastasis. Nature.

[29] Nakata B, Fukunaga S, Noda E, Amano R, Yamada N, Hirakawa K (2008). Chemokine receptor CCR7 expression correlates with lymph node metastasis in pancreatic cancer. Oncology.

[30] Tang RF, Wang SX, Peng L, Wang SX, Zhang M, Li ZF, Zhang ZM, Xiao Y, Zhang FR (2006). Expression of vascular endothelial growth factors A and C in human pancreatic cancer. World J Gastroenterol.

[31] Kurahara H, Takao S, Maemura K, Shinchi H, Natsugoe S, Aikou T (2004). Impact of vascular endothelial growth factor-C and -D expression in human pancreatic cancer: its relationship to lymph node metastasis. Clin Cancer Res.

[32] Wehler T, Wolfert F, Schimanski CC, Gockel I, Herr W, Biesterfeld S, Seifert JK, Adwan H, Berger MR (2006). Strong expression of chemokine receptor CXCR4 by pancreatic cancer correlates with advanced disease. Oncol Rep.

[33] Niu ZX, Fei LM, Wang CL (2009). Expression of CXCL12-CXCR4 and its association with angiogenesis in pancreatic cancer. Zhonghua Zhong Liu Za Zhi.

[34] Neel NF, Schutyser E, Sai J, Fan GH, Richmond A (2005). Chemokine receptor internalization and intracellular trafficking. Cytokine Growth Factor Rev.

